# Comparison of Polydopamine-Coated Mesoporous Silica Nanorods and Spheres for the Delivery of Hydrophilic and Hydrophobic Anticancer Drugs

**DOI:** 10.3390/ijms20143408

**Published:** 2019-07-11

**Authors:** Anna-Karin Pada, Diti Desai, Kaiyao Sun, Narayana Prakirth Govardhanam, Kid Törnquist, Jixi Zhang, Jessica M. Rosenholm

**Affiliations:** 1 Pharmaceutical Sciences Laboratory, Faculty of Science and Engineering, Åbo Akademi University, BioCity (3rd floor), Tykistökatu 6A, FI 20520 Turku, Finland; 2 Key Laboratory of Biorheological Science and Technology, Ministry of Education, College of Bioengineering, Chongqing University, No. 174 Shazheng Road, Chongqing 400044, China; 3 Cell Biology, Faculty of Science and Engineering, Åbo Akademi University, BioCity (2nd floor), Tykistökatu 6A, FI 20520 Turku, Finland; 4 Minerva Foundation Institute for Medical Research, Biomedicum Helsinki, Tukholmankatu 8, 00290 Helsinki, Finland

**Keywords:** mesoporous silica nanoparticles, polydopamine, nanorods, drug delivery system, nanocarrier, cytotoxicity, sustained drug release, shape effect

## Abstract

Mesoporous silica nanoparticles (MSNs) have been widely studied as drug delivery systems in nanomedicine. Surface coating of MSNs have enabled them to perform efficiently in terms of bioavailability, biocompatibility, therapeutic efficacy and targeting capability. Recent studies have suggested the use of polydopamine (PDA) as a facilitative coating for MSNs that provides sustained and pH-responsive drug release, owing to the adhesive “molecular-glue” function of PDA. This further endows these hybrid MSN@PDA particles with the ability to carry large amounts of hydrophilic drugs. In this study, we expand the feasibility of this platform in terms of exploring its ability to also deliver hydrophobic drugs, as well as investigate the effect of particle shape on intracellular delivery of both a hydrophilic and hydrophobic anticancer drug. MSN@PDA loaded with doxorubicin (hydrophilic) and fingolimod (hydrophobic) was studied via a systematic in vitro approach (cellular internalization, intracellular drug distribution and cytotoxicity). To promote the cellular uptake of the MSN@PDA particles, they were further coated with a polyethylene imine (PEI)-polyethylene glycol (PEG) copolymer. Drug-loaded, copolymer-coated MSN@PDA showed effective cellular uptake, intracellular release and an amplified cytotoxic effect with both doxorubicin and fingolimod. Additionally, rods exhibited delayed intracellular drug release and superior intracellular uptake compared to spheres. Hence, the study provides an example of how the choice and design of drug delivery systems can be tuned by the need for performance, and confirms the PDA coating of MSNs as a useful drug delivery platform beyond hydrophilic drugs.

## 1. Introduction

The implementation of nanotechnology in medicine for diagnostics and therapeutics to manage biological systems on the molecular scale have led to the establishment of the interdisciplinary field of nanomedicine [1]. Cancer nanotechnology, the main indication for nanomedical solutions so far, has seen the development of a vast array of nano-systems for early diagnosis and treatment against different types of cancers, and has exhibited enhanced therapeutic effects and lowered toxicity compared to conventional therapies [2,3,4].

Amongst these, mesoporous silica nanoparticles (MSNs) have been frequently used in cancer nanomedicine as drug delivery systems, imaging probes and theranostic agents due to their superior physicochemical characteristics [5,6,7]. Their mesoporous structure, high surface area, tunable pore diameter and pore volumes, flexible and selective surface modification, and enhanced loading of multiple drug cargoes have boosted the development of MSN-based nanocarrier systems [8,9,10]. Furthermore, functionalized MSNs can assist in stimuli-triggered, targeted and controlled drug release through, e.g., changes in pH, light intensity, temperature, ultrasound, the presence of specific enzymes or complementary oligonucleotides [11,12,13,14]. These features highlight the possibility of targeted drug delivery via controlled release of drugs at diseased sites in the body, which is one of the main aims of nanomedical carrier systems.

In one of our previous studies, we put forward the possibility of enhancing the potential of polydopamine (PDA) as a drug delivery agent in combination with MSN [15]. Polydopamine, derived from the mussel adhesive protein-inspired precursor dopamine, exhibits flexible design options with regard to the functionalization of biomaterials because the polymerization can take place under mild conditions and on most surfaces, and further modification of the formed polymeric function is straightforward and versatile [15]. To introduce a structural component to the system, MSNs are the ideal substrate for PDA functionalization and for combining structure with function. Thus, to create such a system, we constructed MSN@PDA hybrid particles by coating of amino-functionalized MSN-NH_2_ with PDA via oxidant induced surface polymerization of dopamine. The presence of the catechol groups in PDA also allowed the construction of a catechol-metal drug coordination system for a pH-responsive drug release, and therefore, Fe^3+^ (MSN@PDA-Fe) and Zn^2+^ (MSN@PDA-Zn) were constructed for this purpose and compared. The investigation of the controlled release behavior based on coordination bonding (catechol-metal and metal-drug) was conducted. We found that the PDA coating improved both the drug loading capacity and drug release properties for hydrophilic drug cargo in terms of providing a sustained and acid-triggered release [15].

In this follow-up study, to evaluate the shape effect we applied the same functionalization regime for rod-shaped MSNs [16] and compared the performance of these as drug carrier systems in vitro on cancer cell cultures (MDA-MB-231 and ML-1) to the earlier constructed spherical MSN@PDA particles. Additionally, we evaluated the feasibility of these nanocarrier systems to deliver a water soluble/hydrophilic drug, doxorubicin (DOX) and a poorly soluble/hydrophobic drug, fingolimod (FTY720). FTY720 is an immunomodulatory drug that has shown promising effects for the treatment of diseases associated with inflammation and uncontrolled cell proliferation such as cancer, but the severe off-target effects urgently calls for the development of more selective formulations for achieving successful treatment regimens with this drug [17]. The intracellular uptake of MSN@PDA particles was investigated and compared in human breast cancer (MDA-MB-231) cells, using confocal microscopy and fluorescence activated cell sorting (FACS). The intracellular distribution of DOX-loaded MSN@PDA particles was compared in MDA-MB-231 cells using live cell imaging. The cytotoxicity of empty and DOX-loaded MSN@PDA particles was evaluated and compared in MDA-MB-231 cells, while the cytotoxicity of empty and FTY720-loaded MSN particles was evaluated in human follicular thyroid cancer cells (ML-1). In summary, we evaluated spherical and rod-shaped MSNs modified with PDA as drug carriers compared to free hydrophobic and hydrophilic drugs, and examined how the difference in MSN morphology influences the performance of this promising drug delivery system.

## 2. Results and Discussion

### 2.1. Characterization of the Nanoparticles

**Figure 1:**
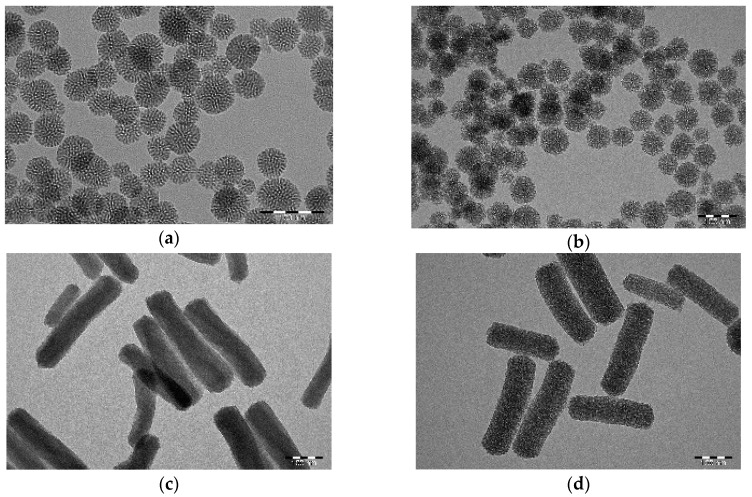
TEM images of (**a**) S-MSN (**b**) S-MSN@PDA (**c**) Rod-MSN (**d**) Rod-MSN@PDA. Scale bar = 100 nm for all images.

PDA-coated spherical (S-MSN) and rod-shaped (R-MSN) particles were synthesized and PDA-modified according to our previously reported protocols [15,16]. To achieve different morphologies, different amounts of aminosilane was used in the syntheses (10 mol% for S-MSN and 1 mol% for R-MSNs) which was also reflected in the differences in zeta potential (Table 1) between uncoated spheres and rods. However, in both cases the exposed amino groups on the surface was sufficient for successful coating of PDA, which is clearly observable in the TEM images (Figure 1). To enhance the dispersability in the complex biological environment and cellular uptake ability, both PDA-modified MSNs were further coated with an in-house produced PEG-PEI copolymer (COP) [18] for cellular studies. The success of each functionalization step was followed by the measurement of hydrodynamic size (DLS) and zeta (ζ) potential (Table 1) to confirm that aqueous dispersability was maintained and aggregation of particles did not occur (DLS). A shift in net surface charge (ζ-potential) further confirmed the alteration in surface properties of the particles after coating. Despite the higher negative net charge resulting from successful PDA-coating, the electrostatic stabilization did not seem sufficient to stabilize, especially in the case of the rod-shaped particles (R-MSN@PDA), as reflected in their high Z-average size and PdI value. Indeed, a decrease in hydrodynamic diameter was observed after COP coating for both particles, thus suggesting enhanced aqueous dispersability via a combination of (mainly) steric and electrostatic stabilization in accordance with our previous study [18].

The intensity weighted particle size distributions of R-MSN and S-MSN, with PDA and COP coating are presented in Figure S1 and S2 (Supplementary Materials) and illustrate the aggregation of R-MSN@PDA without the polymer coating. Thus, in order to prevent aggregation and also to enhance the cellular uptake, both MSN@PDA were coated with COP to increase the colloidal stability of the particles. The success of COP coating can be observed from the size and net surface charge measurements in Table 1. Even though they have lower net charge (ζ-potential), the PEG chains in COP provide a steric component that leads to steric stabilization of the particles [18]. In contrast to S-MSNs (Figure S1), the particle size distribution of R-MSN (Figure S2) changes considerably upon PDA-modification, whereas further coating with COP restores the dispersibility of the particles. However, we note that especially for the rod-shaped MSNs, DLS measurements cannot be regarded as a size measurement due to their non-symmetrical shape; but they are used here as a confirmation of aqueous dispersibility after all functionalization steps. DLS is only applicable for symmetrical/spherical shaped particles for determining particle size; also, this method quite drastically overestimates the diameter of porous particles [19]. Thus, for determining particle size and morphology, TEM imaging was applied instead (Figure 1A,C). The successful coating of PDA could also be clearly detected (Figure 1B,D). The acquired TEM images were further used to assess the particle size distribution using ImageJ. The particle size distribution was analyzed based on size, roundness, and aspect ratio (Figure S3 and S4). The Feret diameter was used as the standard for size measurement and determining size distribution. The ratio of maximum and minimum Feret diameter was analyzed to determine the aspect ratio distribution of the particles. The inverse of the aspect ratio was analyzed to determine the roundness of the particles [20]. The size, aspect ratio, and roundness for S-MSN were 65 nm, 1.2, 0.9, and for R-MSN they were 472.4 nm, 2.42, 0.46, respectively (Figure S3 and S4, Supplementary Materials). The mean aspect ratio of 2.42 for R-MSN affirmed them as short rods, compared to the mean aspect ratio of 1.2 for S-MSN. Based on these characterization results, the COP-coated MSN@PDA were used for further studies with cells.

### 2.2. In Vitro Evaluation of MSN@PDA as a Drug Delivery System

The drug delivery capability of MSN@PDA was investigated based on cellular uptake studies, cytotoxicity assays, and intracellular drug distribution. These results have been evaluated based on the activity of the unloaded and drug-loaded COP-coated S-MSN@PDA and R-MSN@PDA and the corresponding concentrations of the free drug. Doxorubicin (DOX, hydrophilic) and fingolimod (FTY720, hydrophobic) were chosen as the drug models for this study.

DOX is a water soluble (2600 mg/L at 25 °C) potent anticancer drug with a fluorescent hydroxyl-substituted anthraquinone chromophore and a hydrophilic aminoglycosidic side chain used for treating several types of cancers [21]. The DOX mechanism of action consists of two parts. Firstly, it disrupts topoisomerase-II-mediated DNA repair and generates free radicals that damage the cellular membranes, DNA and proteins. Secondly, the oxidation of DOX leads to formation of reactive oxygen species or ROS, which trigger the apoptotic pathways to cell death [22]. The fluorescence is exploited for localizing and studying DNA interactions of DOX. The emission wavelength varies between 560–590 nm depending on the solvent, pH and the drug concentration [21].

FTY720 is a poorly soluble (6.9 mg/L as determined using virtual computation software ALOGPS 2.1, drugbank.ca 2019) immunosuppressive drug, usually used for treating multiple sclerosis by inhibiting lymphocyte migration [23,24,25]. FTY720 is a prodrug, hence after administration it is converted to its active form, and acts as a functional antagonist in lymphocytes and downregulates the gene expression [23,24]. The in vitro investigation of FTY720 to inhibit the migration and attenuate the proliferation of follicular thyroid cancer ML-1 cells proved to be successful in studies by Kalhori et al. [26], and met our goals as an efficient drug model on ML-1 cells.

The loading degree of DOX in S-MSN@PDA was around 9 wt%, and in R-MSN@PDA, it was around 11 wt%. The FTY720-loading degree was adjusted to 16 wt% in both MSN@PDA particles. The possible reason for the slight deviation between spheres and rods for DOX (given that the same initial concentration was used) could be due to the difference in the shape and volume of the particles, resulting in a higher loading degree in R-MSN@PDA. Hao et al. [27] also compared the shape effect on drug loading capacity using DOX, and reported that long rod-shaped MSNs typically showed higher drug loading abilities than short rod-shaped MSN and spherical MSNs. This is most likely connected to the longer diffusion path, making desorption slower during the loading process.

#### 2.2.1. Cellular Uptake of MSN@PDA-COP Particles

**Figure 2:**
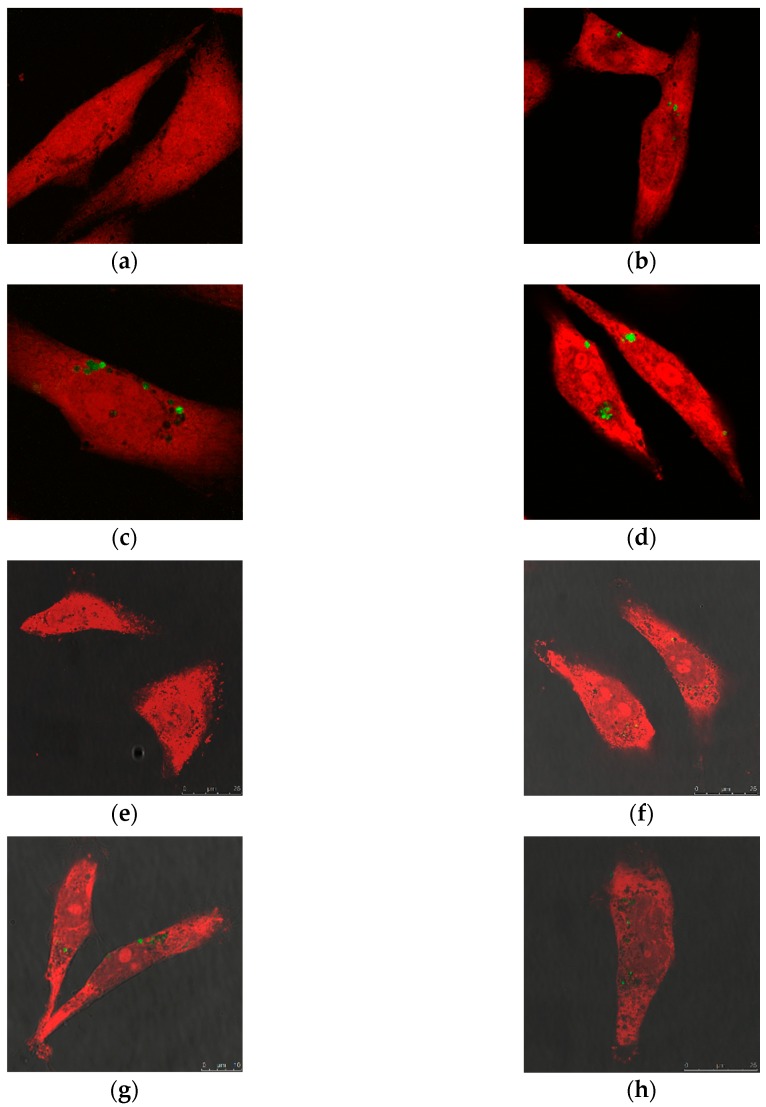
Confocal microscopy images showing endocytosis of S-MSN@PDA (FITC-labelled; green), and R-MSN@PDA (FITC-labelled; green), respectively. (**a**,**e**) control (no particles); (**b**,**f**) 5 µg/mL; (**c**,**g**) 10 µg/mL; and, (**d**,**h**) 25 µg/mL, respectively. The images were taken using 63X oil objective lens after 24 h incubation with MDA-MB-231 cells (PI-stained; red). Scale bar = 25 µm for all images.

Cellular uptake was studied using confocal microscopy and FACS as complementary methods. Confocal microscopy was performed with particle concentrations of 5, 10 and 25 µg/mL. MDA-MB-231 cells (propidium iodide stained, hence, red in color) were incubated with FITC-labeled S-MSN@PDA and R-MSN@PDA (green in color) for 24 h and imaged. Figure 2 shows images of the cells incubated with empty S-MSN@PDA particles and R-MSN@PDA, respectively. The particles (green) can be seen inside the vesicles (black dots) in the cells (red) very clearly, which indicates that the particles are taken up by endocytosis. With higher concentrations of particles, more green dots are visible in the cells, indicating that there is a higher uptake with higher concentrations of particles. A similar pattern is observed in both S-MSN@PDA and R-MSN@PDA.

**Figure 3:**
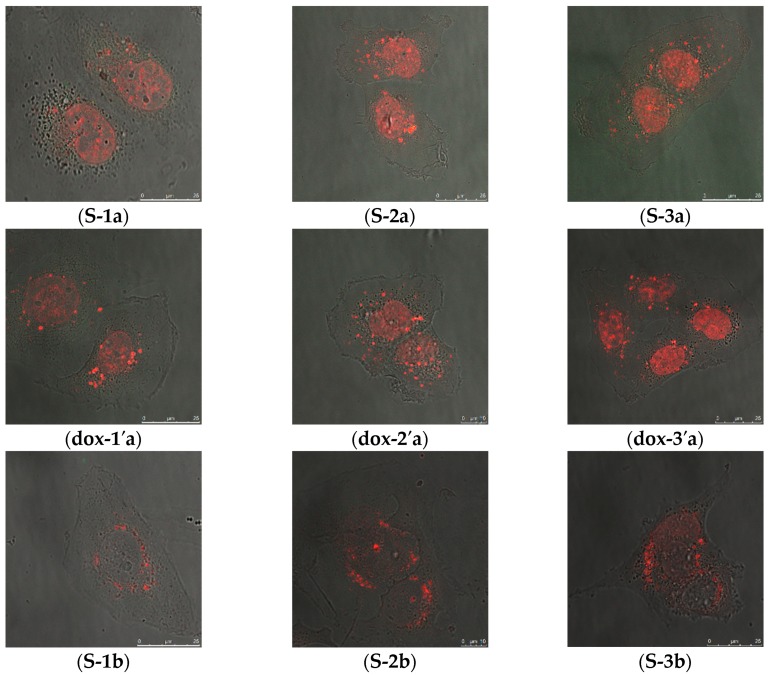

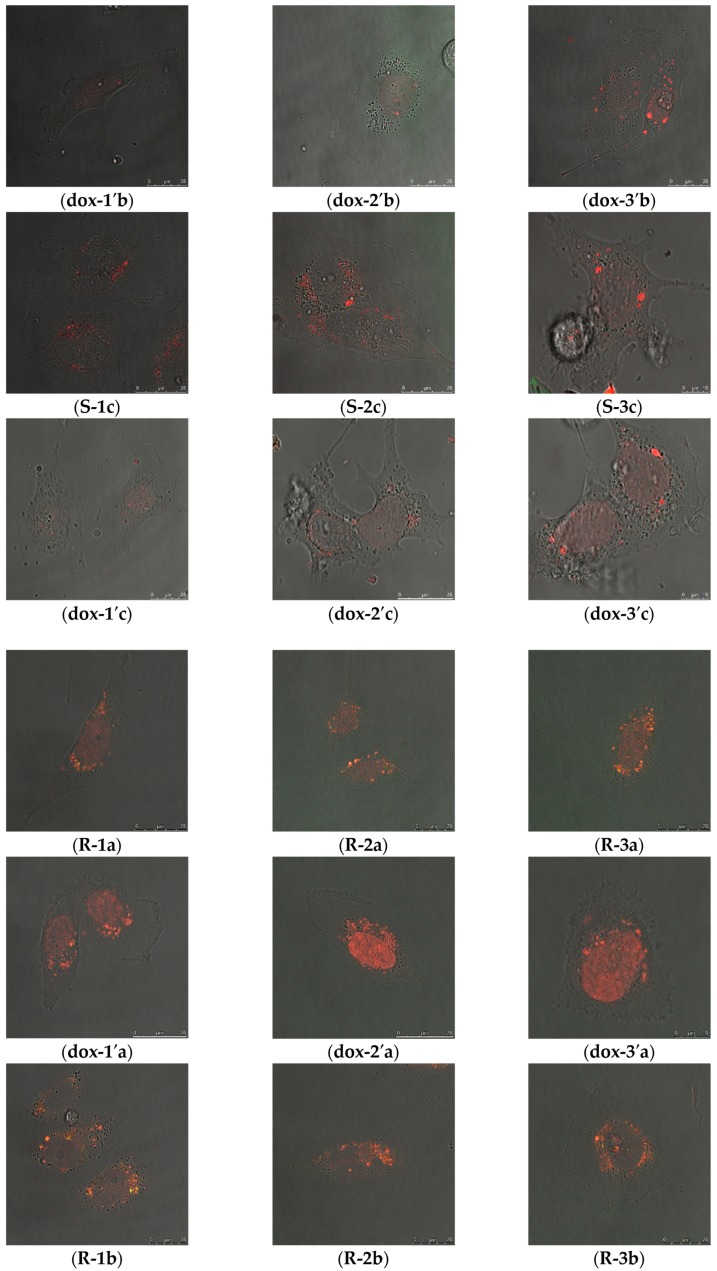

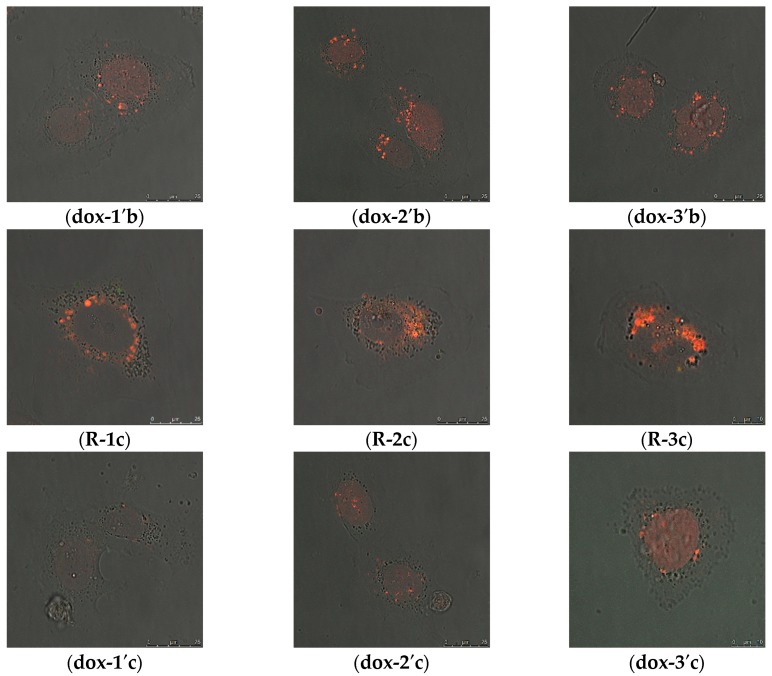
Intracellular distribution of DOX delivered by MSN@PDA-COP particles are represented in the images above in MDA-MB-231 cells over a duration of 24, 48, and 72 h. Images are denoted based on particle type (**R**-rod**, S**-sphere), concentration (**1**-12.5 µg/mL, **2**-18.75 µg/mL and **3**-25 µg/mL) of DOX-loaded particles and time (**a**-24 h, **b**-48 h and **c**-72 h). The prime (**‘**) indicates the corresponding concentrations of free DOX. Live cell images of MDA-MB-231 cells incubated with DOX-loaded particles and free DOX for (**a**) 24 h, (**b**) 48 h and (**c**) 72 h (red color indicates DOX). The images were taken using 63X oil objective lens. Scale bar = 25 µm for all images.

For FACS measurements, the cells were incubated with FITC-labelled S-MSN@PDA (10, 25, and 50 µg/mL) and lower doses R-MSN@PDA (5, 10, and 25 µg/mL). Figure 3 shows a cytometry histogram showing endocytosis of the S-MSN@PDA and R-MSN@PDA. The uptake by the cells was very low for the 5 µg/mL concentration of S-MSN@PDA, which may be due to the quenching effect of PDA. PDA possesses a strong fluorescence quenching ability on a wide spectrum of fluorescent dyes due to its intrinsic light absorbing properties as well as the photo-induced electron transfer process. To better visualize and compare the endocytosis properties of particles by both fluorescence microscopy and flow cytometry, high dosages of particles were employed. In the case of R-MSN@PDA, very high particle concentrations started to exhibit cytotoxicity, as observed from the cell viability tests (Figure 6). Hence, FACS was performed using higher concentrations of S-MSN@PDA than R-MSN@PDA.

The flow cytometry also shows that the particles are taken up by the cells, as seen previously in the confocal microscopy images (Figure 2). The uptake increases with higher concentrations for both particle types. Thus, the uptake study with FACS confirms the phenomenon seen in the microscopy images. The uptake was calculated from the geometrical mean value obtained during FACS and the values are as shown in Table 2.

We note that the uptake measured with FACS may not be as efficient as could be expected based on earlier studies involving COP as surface coating for enhancing cellular internalization [28], which may be associated with the above-mentioned quenching effect of PDA. Thus, the presence of a PDA coating would reduce the fluorescence of the underlying particles, leading to emission of dull-green fluorescence from PDA-modified MSNs. Further, trypan blue treatment was used for quenching the extracellular fluorescence from non-internalized particles for more accurate FACS measurements and trypan blue reduces the fluorescence signal from dull-green emitters [29]. Thus, the fluorescence is semi-quantitative at best, and quantification is further complicated by particle incorporation [30]. However, it can be observed from the FACS study (Figure S5 in Supplementary Materials) that the cellular uptake of R-MSNs was more efficient than S-MSNs. Huang et al. [31] also showed in their study that rod-shaped MSNs induce higher endocytosis compared to spherical MSNs. The shape of rod-shaped MSN allows them to have a larger contact area with the cell membrane, since the longitudinal axis of the rods interacts with the cell membrane. Rod-shaped MSNs have a larger volume, and therefore needs a shorter time to break through kinetic barriers across the lipid membrane than spherical MSN. Hao et al. [27] studied the endocytosis mechanism of spherical and rod-shaped nanomaterials and found that spherical particles were taken up via the clathrin-mediated endocytosis pathway, whereas the rod-shaped particles were taken up via the caveolae-mediated endocytosis pathway. Hence, the cellular uptake of S-MSN@PDA was possibly through the clathrin pathway, and R-MSN@PDA was through the caveolae pathway leading to different levels and/or kinetics of internalization.

#### 2.2.2. Intracellular Distribution of DOX Delivered by MSN@PDA-COP Particles

The intracellular distribution of DOX-loaded particles in MDA-MB-231 cells over a duration of 24, 48, and 72 h was studied, and DOX-release was studied using live cell imaging. Live cell imaging of cells treated with DOX-loaded S-MSN@PDA and R-MSN@PDA particles was performed using different particle concentrations (12.5, 18.75, and 25 µg/mL) and corresponding concentrations of free DOX. Images of the cells treated with DOX-loaded particles are shown in Figure 3. DOX fluorescence intensity from the DOX-loaded particles (Figure S6, Supplementary Materials) is highest after 24 h of incubation and decreases with longer incubation times, as observed from the live cell images (Figure 3). DOX released from MSN@PDA particles, and the free DOX enter the cell nuclei (Figure 3, 1-3c and 3c’) and initiate the mechanism of action [22]. A similar pattern is observed in both S-MSN@PDA and R-MSN@PDA particles. The fluorescence intensity of DOX was analyzed using LAS AF Lite and a comparison of signal intensities w.r.t. concentrations of S-MSN@PDA and R-MSN@PDA over a duration of 24, 48, and, 72 h (in the Supplementary Materials, Figure S6). The fluorescence intensity increased with higher concentrations of DOX-loaded particles and free DOX (Figure S6). Furthermore, it can be observed that after 24 h of incubation, the fluorescence intensity from MSN@PDA particles is higher than the corresponding concentrations of free drug. This indicates that DOX release from the particles is sustained and gradual. Hence, DOX uptake into the cytoplasm and further into the nucleus, where its mechanism of action is initiated (as stated by Thor, et al. [22]) is slow. DOX release from R-MSN@PDA is even slower compared to the release from S-MSN@PDA. In the case of R-MSN@PDA, the fluorescence intensity is similar to free DOX until 48 h. However, a visible difference can be observed after 72 h (Figure S6c,f). The fluorescence intensities at 48 h (Figure S6b,e) and 72 h (Figure S6c,f) represent the differences between rods and spheres. This outcome supports the possibility of the differences in the pore alignment in spheres (radial) and rods (lateral and helical), which results in a delay in the drug release from the rods compared to the spheres, as studied by Zhang et al. [32], and similar to our observation in the cytotoxicity study of DOX-loaded particles.

#### 2.2.3. Cytotoxicity of Empty and Drug-Loaded MSN@PDA-COP Particles

The cytotoxicity of S-MSN@PDA, R-MSN@PDA, and DOX-loaded MSN@PDA particles was studied using WST-1 in MDA-MB-231 cells. However, it is worth noting that the UV-vis absorption range of PDA is broad and indefinite over 200–1000 nm [33], thus suggesting the possibility of overlapping with WST-1 measurements at 440 nm, which could lead to misinterpretation. Hence, an alternative technique, crystal violet assay, was used for comparative test results and for neutrally assessing the cytotoxicity of MSN@PDA particles. The cytotoxicity of DOX-loaded MSN@PDA particles using this assay was studied in MDA-MB-231 cells, while the cytotoxicity of FTY720-loaded MSN@PDA particles was studied in ML-1 cells.

**Figure 4:**
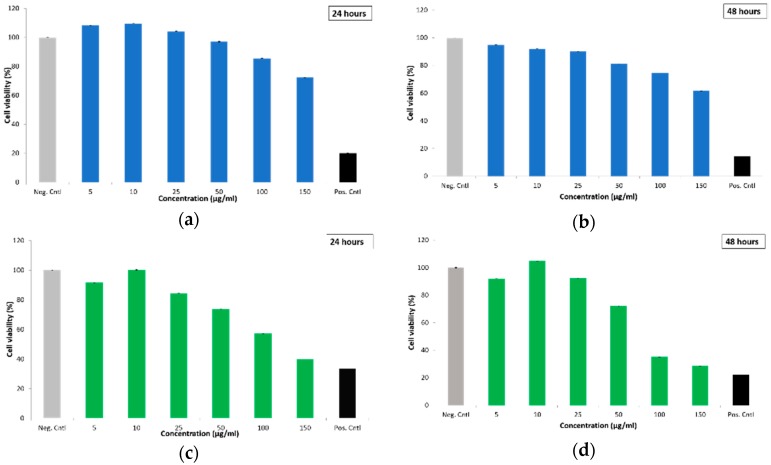
Cell viability of MDA-MB-231 cells as represented using WST-1 assay against the cytotoxic effect of empty MSN@PDA particles, i.e., S-MSN@PDA (**a**,**b**) and R-MSN@PDA (**c**,**d**) at 24 and 48 h of incubation, respectively. Negative control indicates untreated cells, while positive control indicates cells treated with 0.1% Triton X-100. The measurements were performed on a plate reader.

The cytotoxicity of empty and DOX-loaded particles was first determined by WST-1 assay. MDA-MB-231 cells were treated with empty MSN@PDA particles (spheres and rods) for 24 and 48 h with different concentrations (5, 10, 25, 50, 100, 150 µg/mL). Untreated cells as negative control and cells treated with 0.1% Triton X-100 as positive control were also investigated. Cytotoxicity of S-MSN@PDA at lower concentrations (5, 10, 25 µg/mL) was lower than in the negative control (Figure 4a,b). In Figure 4c, it can be observed that the R-MSN@PDA are more toxic than the spherical particles, for which concentrations up to 25 µg/mL show minimal or zero toxicity. Higher concentrations (50, 100, 150 µg/mL) are observed to be highly toxic to the cells after 48 h of incubation (Figure 6d). Huang et al. [31] found similar results in their study, where spherical MSN and long and short rod-shaped MSN were compared. Rod-shaped MSN are easily taken up by endocytosis compared to spherical MSN [31], which we also observed in our FACS measurements (please see Supplementary Materials). Hao et al. [27] also found that rod-shaped MSN has a greater impact on cell functions, such as cell proliferation, apoptosis, cytoskeleton formation, adhesion, and migration, compared to spherical MSNs which corroborates our observations of higher cytotoxicity.

**Figure 5:**
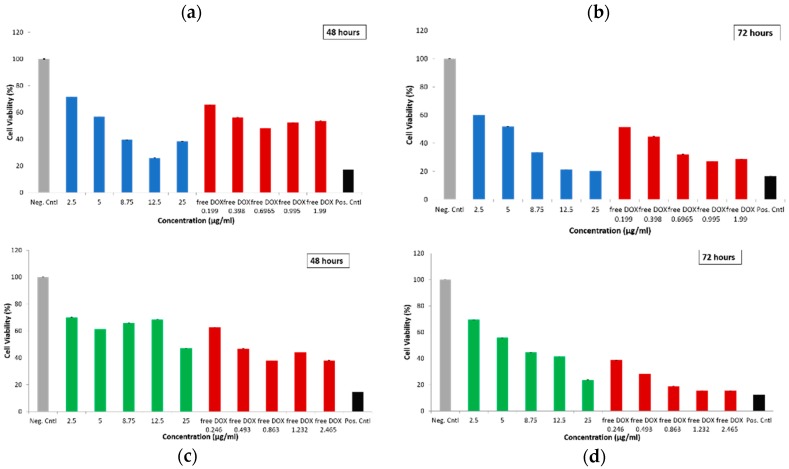
Cell viability of MDA-MB-231 cells as represented using WST-1 assay against the cytotoxic effect of DOX-loaded particles, i.e., DOX-loaded S-MSN@PDA (**a**,**b**), DOX-loaded R-MSN@PDA (**c**,**d**), and corresponding concentrations of free DOX with MDA-MB-231 cells at 48 and 72 h of incubation, respectively. Negative control indicates untreated cells, while positive control indicates cells treated with 0.1% Triton X-100. The measurements were performed on a plate reader.

Next, the cells were treated with DOX-loaded S-MSN@PDA and R-MSN@PDA particles, as well as with corresponding concentrations of free DOX. WST-1 was performed after the cells had been incubated with the DOX-loaded particles and free DOX for 48 and 72 h, respectively. The cytotoxicity of DOX-loaded S-MSN@PDA is higher than the corresponding concentrations of free DOX after 48 and 72 h, as seen in Figure 5a,b. This indicates that S-MSN@PDA might be a suitable carrier for intracellular drug delivery. In the case of R-MSN@PDA, the cytotoxicity is approximately similar to free DOX. This could be due to the alignment of the pores in the rod-shaped MSNs. The lateral and helical alignment of the porous network of R-MSN@PDA, compared to the radial alignment of porous network of S-MSN@PDA, extends the diffusion path for incorporated drug molecules, and as a consequence, the duration of drug release from R-MSN@PDA particles [32] (Supplementary Materials, Figure S7 and S8). Another possible reason could be the overlapping of absorbance wavelengths of PDA (200–1000 nm) and WST-1 (440 nm) as shown by Nieto et al. [33], leading to different interpretations.

**Figure 6:**
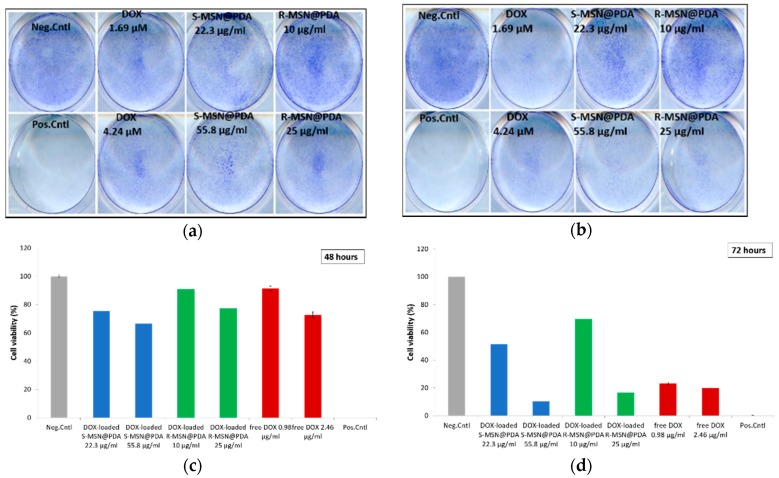
Cell viability of MDA-MB-231 cells as represented using crystal violet assay against the cytotoxic effect of DOX-loaded MSN@PDA particles. The top row (**a**,**b**) shows the scanned images of MD-MBA-231 cells treated with DOX-loaded particles and free DOX (2 repetitions). The bottom row (**c**,**d**) show the cytotoxicity of DOX-loaded S-MSN@PDA, R-MSN@PDA and corresponding concentrations of free DOX with MD-MBA-231 cells at 48 and 72 h of incubation as analyzed using the Colony Area plugin in ImageJ. Negative control indicates untreated cells, while positive control indicates cells treated with 0.1% Triton X-100.

The cytotoxicity of DOX-loaded particles was also determined by crystal violet assay. MDA-MB-231 cells were treated with DOX-loaded particles and with corresponding concentrations of free DOX for 48 and 72 h. The particle concentrations used were different and cannot be directly related to the particle concentrations used for WST-1 assay. For the S-MSN@PDA, the concentrations used were 22.3 and 55.8 µg/mL and for R-MSN@PDA, the concentrations used were 10 and 25 µg/mL. The free DOX concentrations used were 1.69 and 4.24 µM. The plates were scanned for images and were analyzed using the Colony Area plugin in ImageJ. The percentage of cell viability was calculated from the intensity of the crystal color obtained with Colony Area. The crystal violet assay results (Figure 6) resemble a similar pattern w.r.t. WST-1 assay (Figure 5). The efficacy of DOX is higher with higher particle concentrations and greater than free DOX, as observed after 72 h (Figure 6b). The effect of DOX is greater with the S-MSN@PDA compared to the R-MSN@PDA particles. These results further support the probability that a longer duration could be needed for DOX release from R-MSN@PDA particles. However, higher particle concentrations result in higher cytotoxicity. DOX has the ability to bind to DNA-associated enzymes and eventually exhibit a broad range of cytotoxic effects. Unfortunately, treating cancer with DOX often results in severe adverse side effects [34]. These side effects include hematopoietic suppression, nausea, vomiting, extravasation and alopecia. The most feared side effect, however, is cardiotoxicity. The cardiotoxicity can be acute and occur during and within 2–3 days of DOX administration or chronic and appear up to 10–15 years after cessation of chemotherapy. The prognosis for patients that develop myocardial dysfunction is poor; the mortality rate is about 50%. Furthermore, there is no treatment for DOX-induced heart failure and treatment utilizes the standard therapies for congestive heart failure, such as ACE-inhibitors, beta-blockers and loop diuretics for volume management [35,36,37].

**Figure 7:**
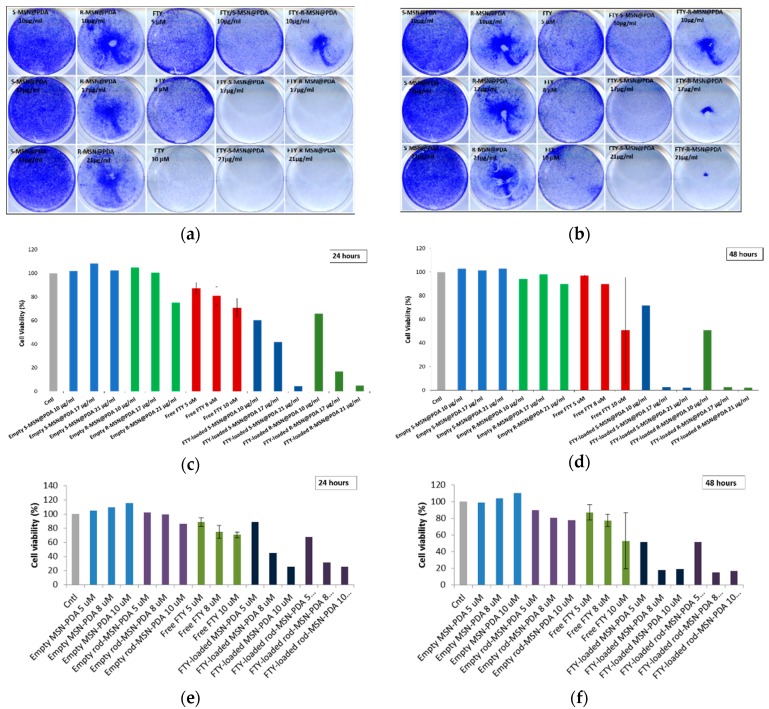
Cell viability of ML-1 cells is represented using crystal violet assay against the cytotoxic effect of FTY720-loaded MSN@PDA particles. The top row (**a**,**b**) shows the well plates used for treating ML-1 cells with FTY720-loaded MSN@PDA particles and free FTY720. The bottom rows show the cytotoxicity of empty and FTY720-loaded S-MSN@PDA, R-MSN@PDA, and corresponding concentrations of free FTY720 in ML-1 cells, analyzed using the Colony Area plugin in ImageJ (**c**,**d**) and plate reader (**e**,**f**) at 24 and 48 h of incubation. Negative control indicates untreated cells, while positive control indicates cells treated with 0.1% Triton X-100.

Cytotoxicity of FTY720-loaded particles was determined by crystal violet assay in ML-1 cells, as they are thyroid carcinoma cells and have shown sensitivity towards FTY720 [26]. ML-1 cells were treated with empty and FTY720-loaded S-MSN@PDA and R-MSN@PDA and corresponding concentrations of free FTY720 (5, 8, and, 10 µM). Two comparative methods for calculation were used, i.e., colony counting and absorbance measurements. The plates were scanned and analyzed using the Colony Area plugin in ImageJ, and absorbance was measured at 590 nm using a plate reader. Viability percentages were calculated for both methods. The results obtained were compared as shown in Figure 7. Empty particles show little (R-MSN@PDA; Figure 7b,d) to zero (S-MSN@PDA) signs of cytotoxicity from both methods of measurement. However, FTY720-loaded MSN@PDA particles induce toxicity, thus, it can be deduced that the increased cytotoxicity is due to FTY720. After 24 and 48 h of incubation, the activity of FTY720-loaded MSN@PDA particles is especially distinguishable at higher concentrations (8 µM and 10 µM) compared to free FTY720. Similar patterns can be observed between S-MSN@PDA and R-MSN@PDA. The data acquired and analyzed from both methods concur, thus, indicating an enhanced therapeutic effect and drug delivery efficacy of the nanocarrier system over the free drug.

In summary, alternative and complementary techniques such as the WST-1 and the crystal violet assay for investigating cytotoxicity aided in the interpretation despite slight variations in the cytotoxic activity of the particles (spheres and rods) in different cell lines. The observed differences are most likely due to the impact of particle shape and variations in cellular uptake kinetics between different cell lines [27]. The WST-1 assay results depict a slightly weaker picture of the behavior of the DOX-loaded particles, probably due to the overlapping of absorbance wavelengths between WST and DOX. Nevertheless, as a complementary method, the crystal violet assay results clarify the hypothesis and confirm the efficacy of MSN@PDA as an intracellular drug delivery system.

## 3. Materials and Methods

Materials and chemicals used were purchased from Sigma Aldrich (Darmstadt, Germany), unless otherwise specified. The methods used for the experiments are described below.

### 3.1. Polydopamine Coating of MSN

S-MSN and R-MSNs were synthesized according to previous protocols [15,16]. The PDA coating was processed by oxidant induced surface self-polymerization of dopamine. Sixty mg of particles was dispersed in 30 mL of HEPES buffer (25 mM, pH 7.4). Twelve mg of dopamine hydrochloride was added and sonicated for 5 minu, followed by the addition of 14.4 mg ammonium persulfate. The addition of ammonium persulfate initiated the polymerization reaction, which was left to proceed overnight under stirring. PDA-coated-MSNs were then collected by centrifugation (4000 rpm, 3 min). The solution was washed 3 times with water to remove unreacted dopamine and stored at +4 °C.

### 3.2. Characterization of MSN@PDA Particles

The different particles (S-MSN@PDA and R-MSN@PDA) were characterized by dynamic light scattering measurements (DLS) for size distribution, zeta potential measurements for charge, and transmission electron microscopy (TEM) for morphological acquisition. Samples with particle concentrations of 0.5 mg/mL were prepared for the measurement of the size distribution and zeta potential. The particles were dispersed in HEPES buffer (25 mM, pH 7.4), MES buffer (10 nM, pH 4.5) or in distilled water. The size distributions and zeta potentials were measured using a Zetasizer Nano instrument (Zetasizer Nano ZS, Malvern, UK). The measurements were done at 25 °C. Samples for TEM were prepared by dispersing polydopamine coated and non-coated particles in ethanol. A few drops of the sample were put on copper grids and allowed to dry. TEM images were obtained using JEM-1400 Plus TEM at the University of Turku (Turku, Finland).

### 3.3. Drug Loading and Loading Degree Determination

The particles (S-MSN@PDA and R-MSN@PDA) were loaded with DOX at 50% (*w*/*w*) w.r.t the particles solution in HEPES buffer (25 mM, pH 7.2) at 2mg/mL concentration. The mixture was stirred for 2 h. Post stirring, the particles were coated with a PEG-PEI copolymer (COP) produced by Karaman et al. (2014) in order to minimize the risk of agglomeration of the particles. One hundred percent (*w*/*w*) of COP w.r.t. the particles, was dissolved in HEPES buffer at 2 mg/mL concentration and added to the drug-loaded particles. The mixture was kept under stirring over night for further loading. The solution was centrifuged (13,500 rpm, 8 min) to collect the DOX-loaded-particles. The loading degree of the DOX-loaded-particles was calculated by subtracting the mass of DOX in the supernatant from the total mass of DOX in the initial solution. The concentrations in the solutions were analyzed with UV-Vis spectrophotometer (NanoDrop 2000c, Thermo Fischer Scientific, Waltham, Massachusetts, USA) at the wavelength of 480 nm. A calibration curve for DOX was done in order to facilitate the calculations (Figure S9).

FTY720 is not very stable in aqueous solutions, hence, the drug loading was performed by ultrasonically dispersing 0.5 mg particles in 500 µL of a drug solution with the concentration of 100 µg/mL. The drug was dispersed in HEPES buffer (25 mM, pH 7.2). COP copolymer coating was performed as mentioned earlier for DOX-loaded particles. The solution was then stirred for 30 min before collecting the FTY720-loaded-particles by centrifugation (13,500 rpm, 8 min) and redispersing them in HEPES buffer (25 mM, pH 7.2). The degree of loading of FTY720-loaded particles was determined as mentioned above for DOX-loaded-particles, at a wavelength of 265 nm (loading degree data not included). Following the drug loading, the particles were ready to use for in vitro experiments.

### 3.4. Cell Cultures

The cells used in this experiment were MDA-MB-231 cells, which are triple negative (in terms of gene expression profiling and immunohistochemical expression of oestrogen receptor, human epidermal growth factor receptor and progesterone receptor) human breast cancer cells [38]. ML-1 cells were used for comparison in the assessment of the nanocarriers’ (S-MSN@PDA and R-MSN@PDA) capability as a drug delivery system. ML-1 cells are human follicular thyroid cancer cells [39].

The culture medium for MDA-MB-231 cells and ML-1 cells are the same, i.e., it consists of Dulbecco’s Modified Eagle’s Medium (DMEM) supplemented with 10% (*v*/*v*) of heat inactivated fetal bovine serum (FBS), the antibiotics penicillin and streptomycin (100 IU/mL), the essential amino acid L-glutamine (2 mM) and non-essential amino acids (NEAA). All the cells were incubated under the same conditions, i.e., in 37 °C and 5% CO2 in a humified cell culture chamber.

### 3.5. Cellular Uptake of MSN@PDA

The cellular uptake of S-MSN@PDA and R-MSN@PDA was studied with confocal microscopy and fluorescence activated cell sorting (FACS). MDA-MB-231 cells were used for examining the possible pathway through which the particles were taken up.

#### 3.5.1. Confocal Microscopy

The density of cells used was 40 × 10^3^ cells per well in 2 mL of culture medium and was cultured overnight in 6-well plates on sterilized coverslips. Different concentrations (5, 10 and 25 µg/mL) of S-MSN@PDA and R-MSN@PDA particles were incubated with the cells for 24 h. The cells were fixed using 4% paraformaldehyde (PFA). Propidium iodide (PI) was used for staining the cells. Prolong Gold anti-fade solution was used for mounting the coverslips. Images were acquired at 63× magnification (Leica TCS SP5 Matrix, Leica microsystems, Wetzlar, Germany). The samples were excited at 488 nm (FITC-labelled S-MSN@PDA and R-MSN@PDA) and 543 nm (PI-stained cells), and the emission ranges 500–530 nm (FITC-labelled S-MSN@PDA and R-MSN@PDA) and 580–620 nm (PI stained cells) were used.

#### 3.5.2. Fluorescent Activated Cell Sorting

The density of cells used was 60 × 10^3^ cells per well in 1 mL of culture medium and cultured overnight in 12-well plates. Post culturing, the cells were incubated for 24 h with different concentrations of S-MSN@PDA (10, 25 and 50 µg/mL) and R-MSN@PDA (5, 10 and 25 µg/mL). Three replicates of all concentrations were used. The samples were trypsinized using 400 µL of a 0.25% trypsin-0.2 % EDTA solution and washed with PBS. The cells were treated with 200 µL of trypan blue for quenching the auto fluorescence of the cells [29]. The cells were washed and suspended in PBS. FACS was performed using a flow cytometer (FACS Calibur, Becton Dickinson and company, East Rutherford, New Jersey, USA).

### 3.6. Intracellular Distribution of DOX-Loaded MSN@PDA Particles

The intracellular distribution of DOX-loaded MSN@PDA particles with different concentrations was investigated using live cell imaging. MDA-MB-231 cells were treated with both DOX-loaded S-MSN@PDA and R-MSN@PDA particles and corresponding free DOX concentrations, and compared. The density of cells used were 40 × 10^3^ cells per well in 2 mL media in a glass bottomed 6-well plate and cultured for 24 h. Post culturing, the cells were treated for 24, 48 and 72 h with different concentrations (12.5, 18.75, 25 µg/mL) of DOX-loaded S-MSN@PDA and R-MSN@PDA particles and corresponding concentrations of free DOX. The cell media was replaced with fresh media (without particles) after every 24 h of incubation to get rid of any extracellular particles. Images were acquired using a confocal microscope (Leica TCS SP5 STED, Leica microsystems) after 24, 48 and 72 h of incubation. The cells were kept at 37 °C during the live cell imaging. Excitation/emission wavelength of 488 nm/520–550 nm (FITC-labelled S-MSN@PDA and R-MSN@PDA) and 580–615 nm (DOX) was used. The fluorescence intensity of DOX was analyzed using LAS AF Lite.

### 3.7. Cytotoxicity

The cytotoxicity of the S-MSN@PDA and R-MSN@PDA was evaluated using different concentrations of the particles, both in the absence and presence of DOX and FTY720, respectively. MDA-MB-231 and ML-1 cells were used for examining the cytotoxicity. Crystal violet assay (0.1% in 20% methanol) was performed independently for DOX-loaded and FTY720-loaded particles. WST-1 was performed for empty and DOX-loaded particles. Untreated cells were used as negative control, while cells treated with 0.1% Triton X-100 were used as positive control.

#### 3.7.1. WST-1 for DOX-Loaded Particles

The cytotoxicity of the empty and DOX-loaded particles was examined in MDA-MB-231 cells. The density of cells used were 6 × 10^3^ cells per well in 100 µL of culture media and cultured overnight in 96-well plates. Post culturing, the cells were treated with different concentrations of empty MSN@PDA particles (5, 10, 25, 50, 100, 150 µg/mL), DOX-loaded MSN@PDA particles (2.5 to 25 µg/mL) and corresponding concentrations of free DOX. Four replicates of each concentration were used. Empty MSN@PDA particles were incubated for 24 and 48 h, and DOX-loaded MSN@PDA particles were incubated for 48 and 72 h. The cell media was replaced after every 24 h of incubation to fresh media (without particles) to get rid of any extracellular particles. Post treatment, 5µL of WST-1 reagent was added and the samples were incubated for 3 h before the absorbance was measured at 440 nm with a plate reader (Varioskan Flash, Thermo Fischer Scientific).

#### 3.7.2. Crystal Violet for DOX-Loaded Particles

The cytotoxicity of DOX-loaded MSN@PDA and corresponding concentrations of free-DOX was examined in MDA-MB-231 cells. The density of cells used were 50 × 10^3^ cells per well in 1 mL cultured media and cultured overnight in 12-well plates. Post culturing, the cells were treated with different concentrations of DOX-loaded S-MSN@PDA (22.3, 55.8 µg/mL) and R-MSN@PDA (10, 50 µg/mL) and corresponding concentrations of free DOX. The cells were incubated with the particles for 48 and 72 h, and the cell media was changed after every 24 h of incubation. Post treatment, the cells were washed with PBS and fixed with PFA, followed by crystal violet staining, and washing with distilled water. After staining, the well plates were left to dry and scanned using Epson scanner (Epson Perfection V700 PHOTO, Epson, Suwa, Nagano Prefecture, Japan). Images were analyzed using ImageJ plugin, ColonyArea. The intensity of the violet colors was analyzed and compared.

#### 3.7.3. Crystal Violet for FTY720-Loaded Particles

The cytotoxicity of empty and FTY720-loaded MSN@PDA, and corresponding concentrations of free-FTY720 was examined in ML-1 cells. The cells were plated in 12-well plates at a density of 70 × 10^3^ cells per well in 1 mL of culture media and cultured overnight. Post culturing, the cells were treated with empty and FTY720-loaded S-MSN@PDA, R-MSN@PDA and corresponding concentrations of free FTY720. Crystal violet assay was performed as described above.

## 4. Conclusions

The hybrid nanocarrier system, MSN@PDA, was successfully constructed in two different shapes and then coated with a PEG-PEI copolymer for enhanced performance as an intracellular drug carrier. The uptake increased with a higher concentration of particles, which was further confirmed with FACS whereby the uptake of rods was observed to be greater compared to spheres. The intracellular distribution of DOX obtained with the delivery mediated by DOX-loaded MSN@PDA particles indicated intracellular release and nuclear localization of DOX. Furthermore, DOX fluorescence analysis suggested the sustained release and nuclear migration of DOX from the nanocarrier system. The cytotoxicity evaluation of DOX-loaded MSN@PDA in MDA-MB-231 cells suggests that S-MSN@PDA is an efficient intracellular drug delivery vehicle. However, in the case of R-MSN@PDA, the observed cytotoxicity was similar to free DOX, possibly due to the lateral alignment of pores, which delays DOX release compared to the radial alignment of the shorter pores in spheres. This is to be expected, since DOX as a free drug is not limited in its cellular internalization. However, for the more hydrophobic FTY720, the impact of MSN formulation was expected to be more pronounced due to its poor solubility. Indeed, the FTY720-loaded MSN@PDA in ML-1 cells showed a significantly higher cytotoxic effect compared to the free drug. Considering the adverse effects of FTY720 drug treatment, a more localized or targeted delivery at lower doses is of great importance. Hence enhanced therapeutic efficacy from sustained and gradual drug release in vitro was evident in both morphologies of the nanocarrier system. However, in this study there were noticeable differences between rods and spheres, including delayed intracellular drug release in rods compared to spheres due to different pore alignment and length, and superior cellular uptake of rods over spheres due to different exposed surface areas. Therefore, morphology tuning can be applied as a design strategy based on the requirements for the drug delivery system.

## Figures and Tables

**Table 1:** Size and zeta potential of uncoated and polydopamine (PDA)-coated S-MSN and R-MSN with and without further PEG-PEI copolymer (COP) coating.

Particle Type	Z-Average Size (nm) (Measured in DI Water)	Polydispersity Index (PdI)	Zeta Potential (mV) (Measured in HEPES Buffer, 25mM, pH 7.4)
S-MSN	181.7 ± 1.45	0.191	15.3
Rod-MSN	248.73 ± 3.90	0.087	−20.83 ± 1.05
S-MSN@PDA	191.77 ± 5.15	0.269	−13.43 ± 0.31
Rod-MSN@PDA	1678.00 ± 159.45	0.313	−22.93 ± 0.96
S-MSN@PDA-COP	158.80 ± 1.32	0.198	3.91 ± 0.49
Rod-MSN@PDA-COP	219.60 ± 4.92	0.097	5.74 ± 0.39

**Table 2:** The uptake (MFI) of the MDA-MB-231 cells by S-MSN@PDA and R-MSN@PDA at different concentrations (µg/mL) as calculated from FACS.

Particle Type	Concentration (µg/mL)	Uptake by Cells (MFI)
S-MSN@PDA	5	30
	10	40
	25	70
	50	150
R-MSN@PDA	5	40
	10	60
	25	165

## Supplementary Materials

Supplementary materials can be found at https://www.mdpi.com/1422-0067/20/14/3408/s1.

## Abbreviations

MSN	Mesoporous silica nanoparticles
PDA	Polydopamine
DOX	Doxorubicin
FTY720	Fingolimod
MSN-NH_2_	Amino-functionalized MSN
MDA-MB-231	Human breast cancer cells
ML-1	Human follicular thyroid cancer cells
FACS	Fluorescence Activated Cell Sorting
S-MSN	Spherical shaped MSN
R-MSN	Rod shaped MSN
PEG-PEI	Polyethylene glycol-Polyethylenimine
DLS	Dynamic Light Scattering
TEM	Transmission Electron Microscopy
WST	Water Soluble Tetrazolium salt
PI	Propidium Iodide
FITC	Fluorescein isothiocyanate
HEPES	4-(2-hydroxyethyl)-1-piperazineethanesulfonic acid
