# Supplementary material for: Comparison of Polydopamine-Coated Mesoporous Silica Nanorods and Spheres for the Delivery of Hydrophilic and Hydrophobic Anticancer Drugs
Source: Int J Mol Sci. 2019 Jul 11;20(14):3408. doi: 10.3390/ijms20143408 (PMC6679059; doi:10.3390/ijms20143408)
Supplement: Supplementary file 1 [file ijms-20-03408-s001.pdf]

SUPPLEMENTARY MATERIALS to article:

Comparison of polydopamine-coated mesoporous silica nanorods and spheres for the delivery of hydrophilic and hydrophobic anticancer drugs

Anna-Karin Pada[1], Diti Desai[1], Kaiyao Sun[4], Naranaya Prakirth Govardhanam[1], Kid Törnquist[2, 3], Jixi Zhang[4,*] and Jessica M. Rosenholm[1*]

Characterization of nanoparticles

The intensity weighted particle size distribution of S-MSN and R-MSN particles, with polydopamine (PDA) and PEG-PEI copolymer (COP6) coating are as shown in Fig S1 and S2, respectively.

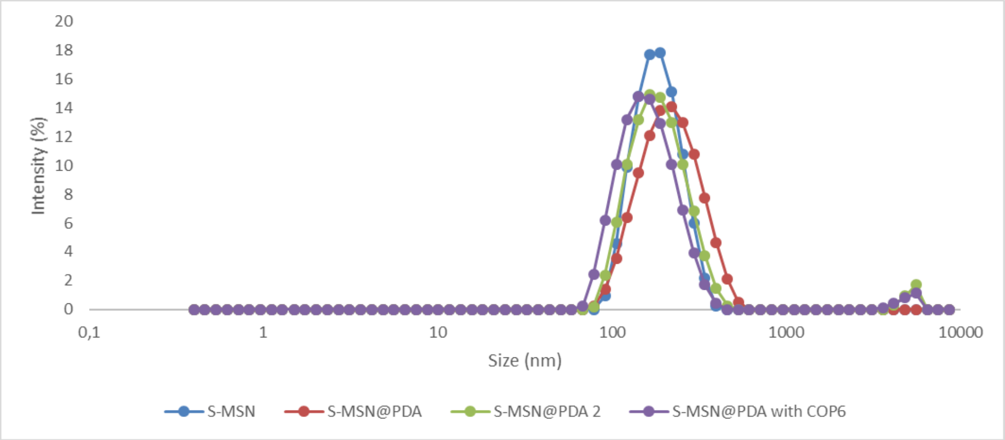

Figure S1. Intensity weighted particle size distributions of S-MSN, with PDA-coating and COP6-coating. Zheng, X., et al (2015) provided the intensity distribution of S-MSN with PDA-coating and COP6-coating

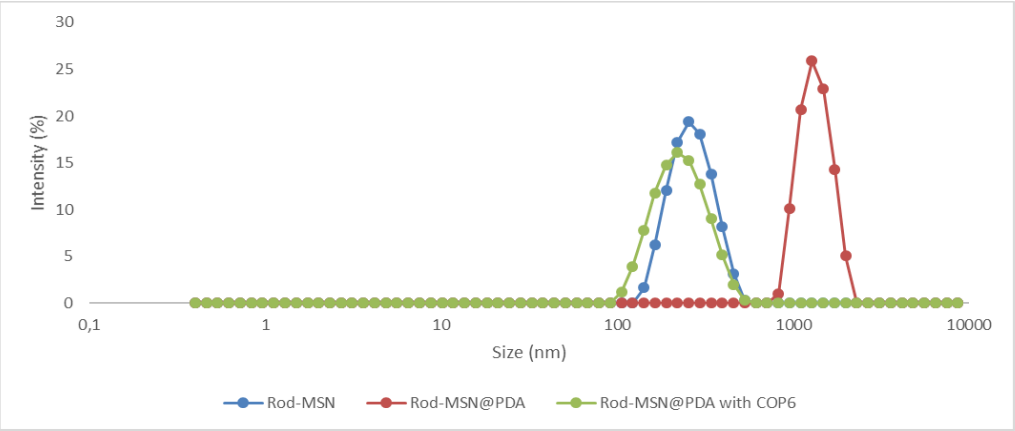

Figure S2. Intensity weighted particle size distributions of R-MSN, with PDA-coating and COP6-coating. After COP6-coating, the redispersibility of the R-MSNs were restored.

Particle size distribution

The particle size distribution was analyzed using Analyze Particles method in ImageJ (Ferreira, T., & Rasband, W., 2012) from 199 particles of S-MSN (Figure S3 a, b, c) and from 185 particles of R-MSN (Figure S4 a, b, c). The surface plot (Fig S3d and S4d) was plotted using 3D-scatter plot from Origin 2019 (Academic). The particles distribution was analyzed based on size, roundness, and aspect ratio. Feret diameter was used as the standard for size measurement and determining size distribution. The ratio of maximum and minimum Feret diameter was analyzed to determine the aspect ratio distribution of the particles. Inverse of the aspect ratio was analyzed to determine the roundness of the particles. The size, aspect ratio, roundness for S-MSN was 65 nm, 1.2, 0.9, and for R-MSN was 472.4 nm, 2.42, 0.46, respectively.

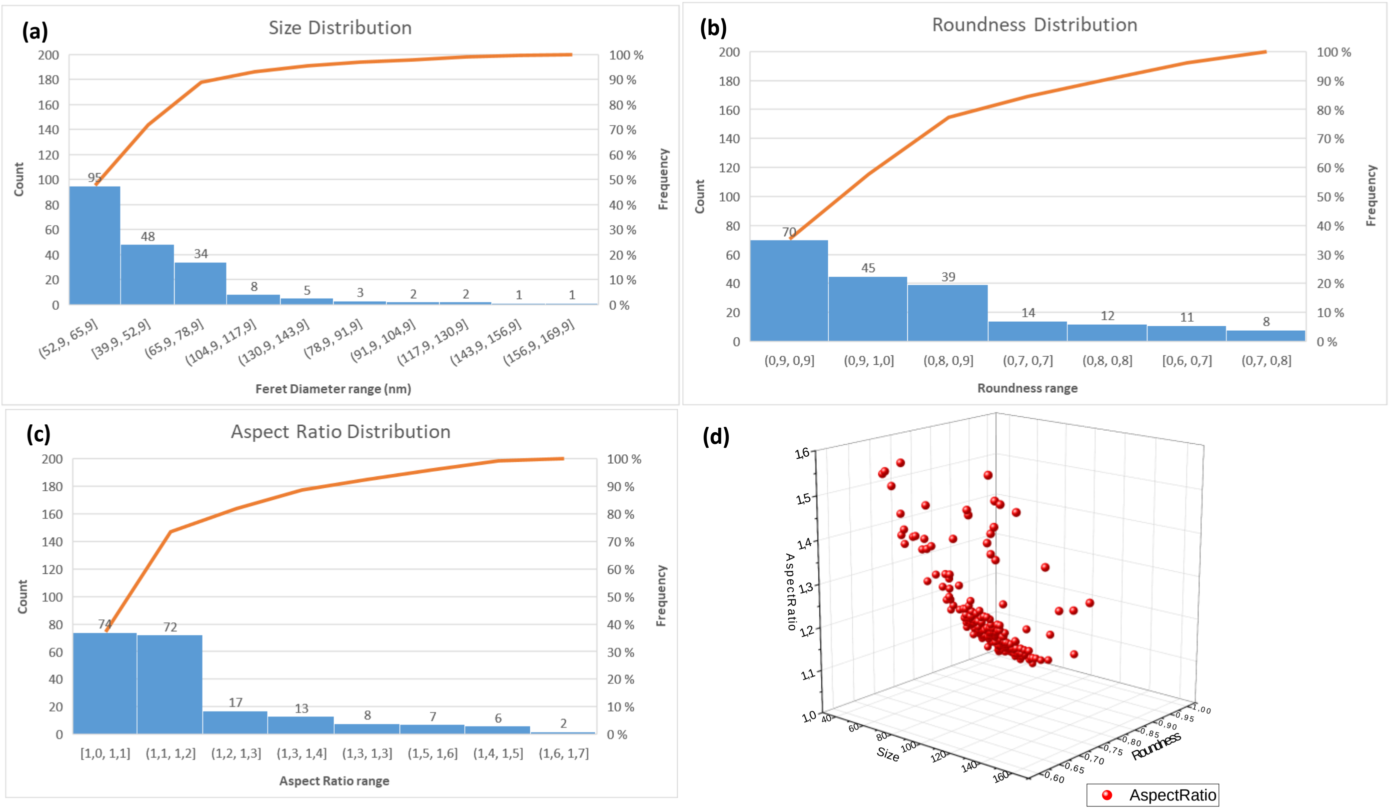

Figure S3. Pareto charts represent the distribution of size (**a**), roundness (**b**), and aspect ratio (**c**) in descending order of frequency, with a cumulative line graph representing the percentage of total S-MSN particles analyzed. The particle size distribution of S-MSN is represented with size, roundness and aspect ratio using 3D-scatter plot (**d**).

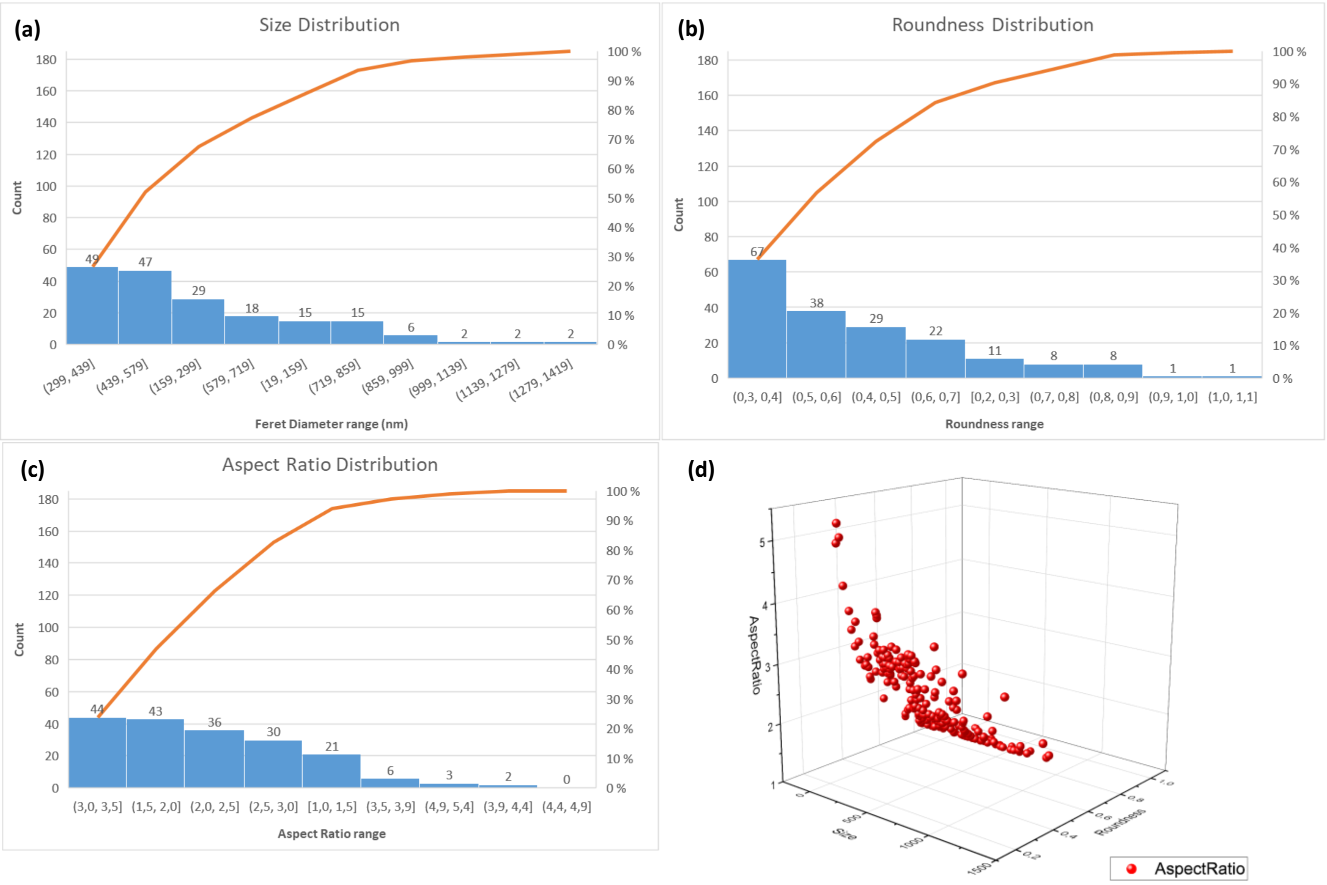

Figure S4. Pareto charts represent the distribution of size (**a**), roundness (**b**), and aspect ratio (**c**) in descending order of frequency, with a cumulative line graph representing the percentage of total R-MSN particles analyzed. The particle size distribution of R-MSN is represented with size, roundness and aspect ratio using 3D-scatter plot (**d**). A mean aspect ratio of 2.42 for R-MSN affirm them as short rods.

Cellular uptake of MSN@PDA-COP particles

For FACS measurements, the cells were treated with FITC-labelled S-MSN@PDA (10, 25, and 50 µg/ml) and R-MSN@PDA (5, 10, and 25 µg/ml). The cytometry histograms show endocytosis of the S-MSN@PDA and R-MSN@PDA at different concentrations. The uptake by the cells was very low for 5 µg/ml concentration of S-MSN@PDA, while for R-MSN@PDA, higher particle concentrations exhibited cytotoxicity as observed from the cytotoxicity tests (Figure 4 in the manuscript). Hence, FACS was performed using higher concentrations of S-MSN@PDA.

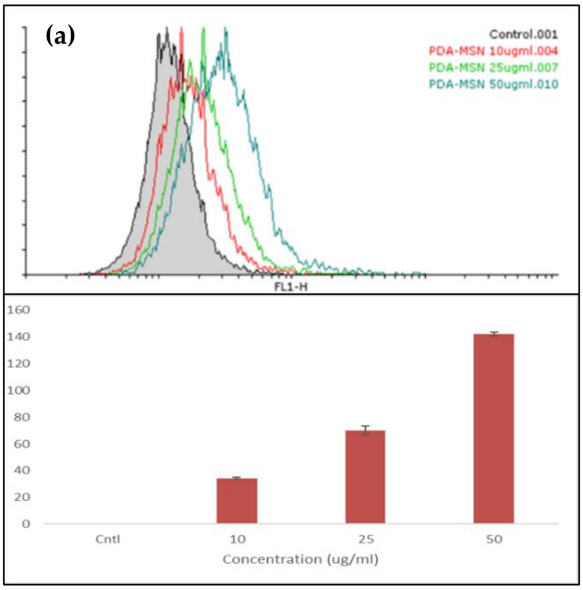

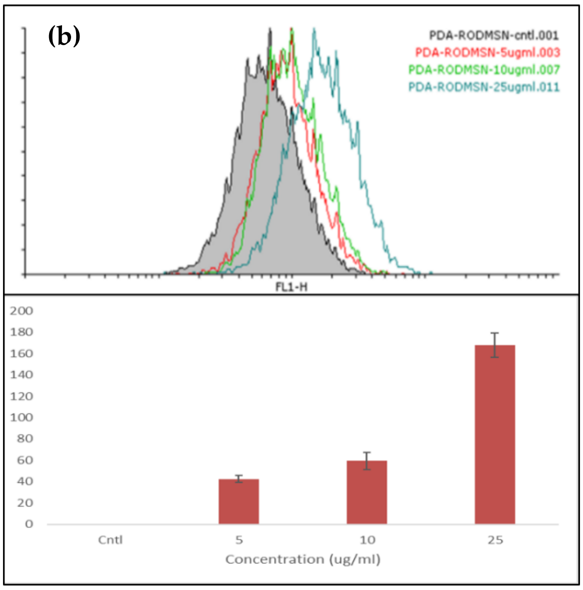

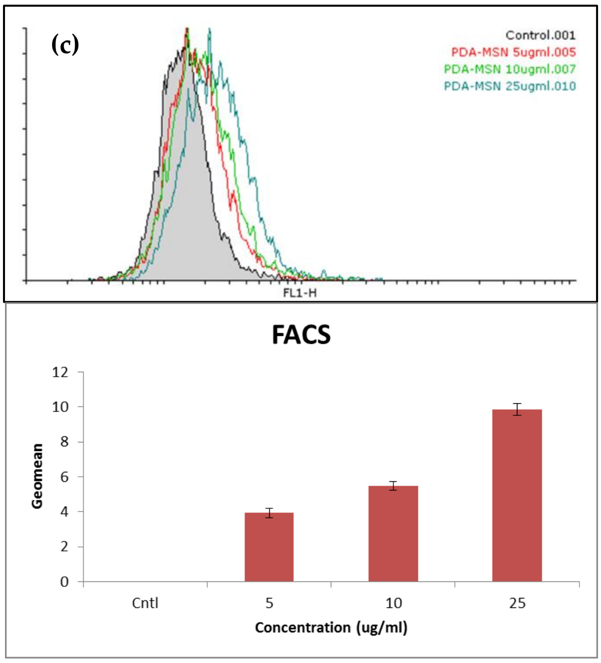

Figure S5. Flow cytometry histogram and graph of S-MSN@PDA (a) showing endocytosis of 10, 25 and 50 µg/ml after incubation for 24 hours with MDA-MB-231 cells. Flow cytometry histogram and graph of R-MSN@PDA & S-MSN@PDA (b & c) showing endocytosis of 5, 10 and 25 µg/ml after incubation for 24 hours with MDA-MB-231 cells. The y-axis of the graphs represents the mean fluorescence intensity (MFI) of the internalized particles.

Intracellular distribution of DOX delivered by MSN@PDA-COP particles

The fluorescence intensity of DOX was analyzed using LAS AF Lite and a comparison of signal intensities, with regard to the concentrations of S-MSN@PDA and R-MSN@PDA over a duration of 24, 48, and 72 hours.

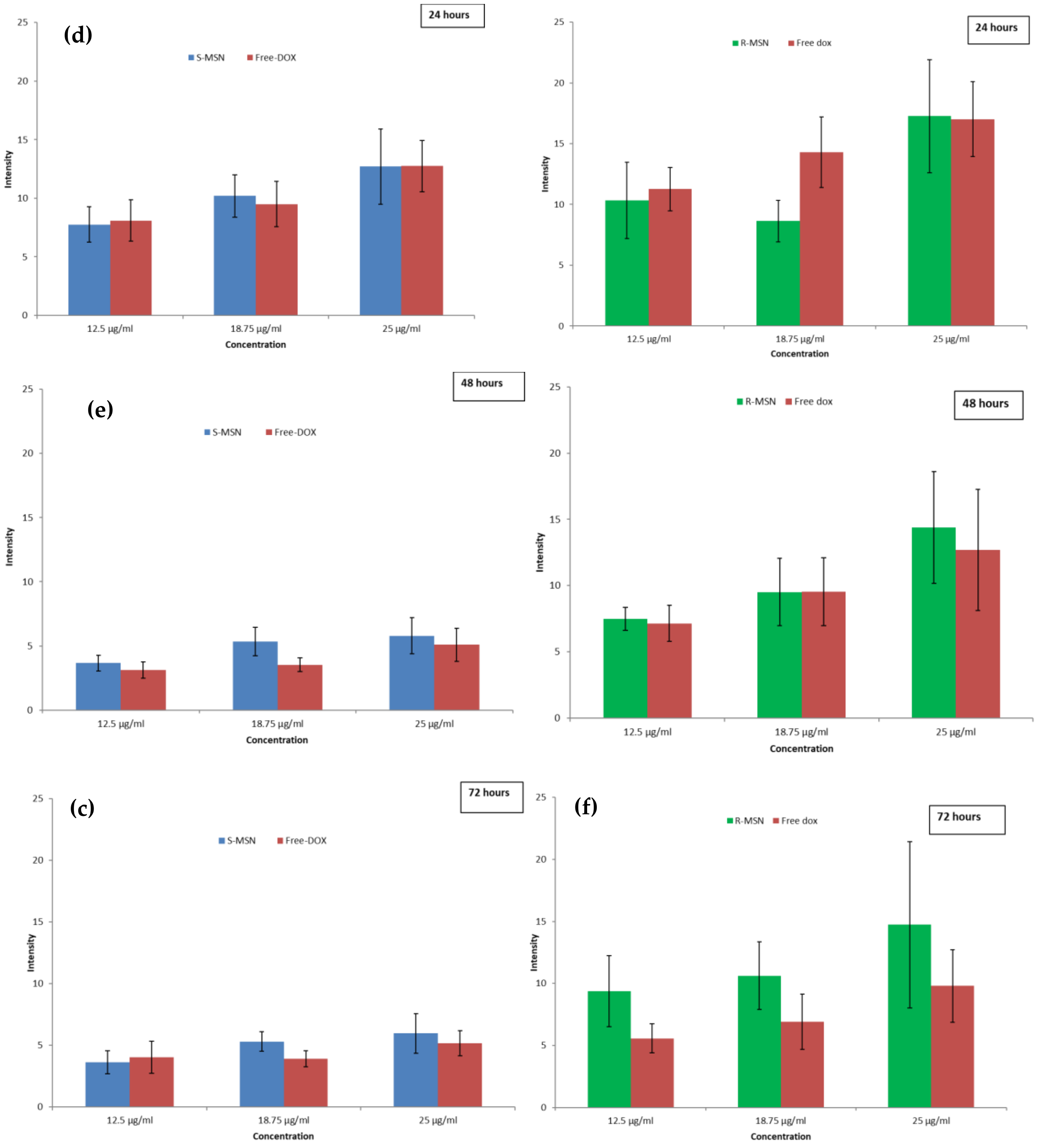

Figure S6. Fluorescence intensity of DOX in MDA-MB-231 cells after incubation with S-MSN@PDA for (a) 24 hours, (b) 48 hours, (c) 72 hours and R-MSN@PDA for (d) 24 hours, (e) 48 hours, and (f) 72 hours.

pH-dependent release of DOX

The drug release from S-MSN@PDA and R-MSN@PDA particles was examined in HEPES buffer (25 nM, pH 7.2) and MES buffer (10 mM, pH 4.5), as represented in figures S8 and S9.

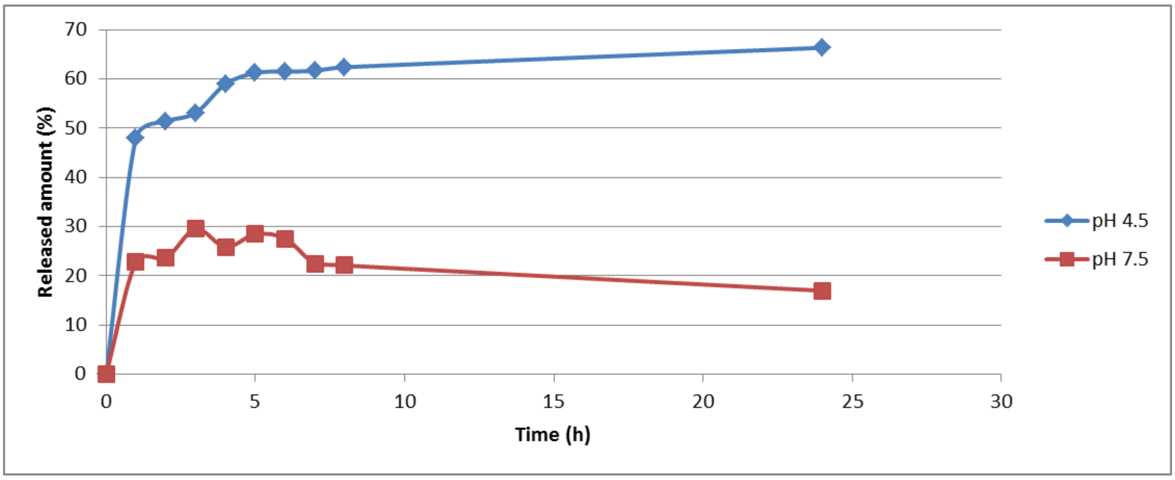

Figure S7. DOX release profiles as a function of time for DOX-loaded S-MSN@PDA particles under acidic and neutral pH conditions.

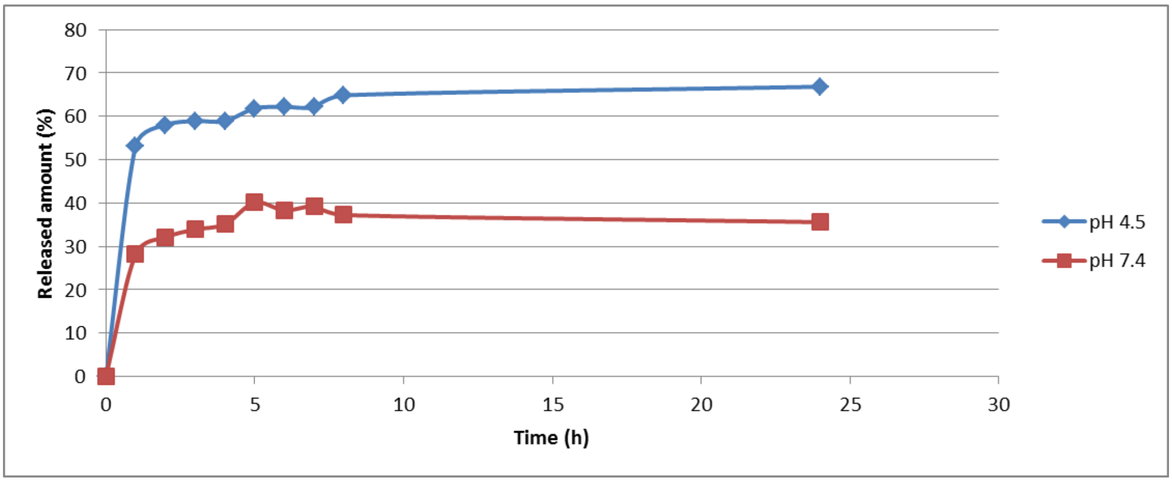

Figure S8. DOX release profiles as a function of time for DOX-loaded R-MSN@PDA particles under acidic and neutral pH conditions.

The drug release from the particles is pH dependent and sustained as observed from the above results. The release is faster in a lower pH than in a higher pH, as expected. A burst release after 1 hour can be observed at both pH 7.2 and pH 4.5, but it is more pronounced at pH 4.5. For the spherical particles (S-MSN@PDA) there is a slow increase in the release until around 20 %, and 60 % of the drug has been released after 24 hours at pH 7.2 and pH 4.5 respectively [1]. The drug release from the rod-shaped particles show similar patterns as for the spherical, with a burst release after 1 hour and then a slow increase until around 30 %, and 60 % of the drug has been released at pH 7.2 and pH 4.5 respectively. The fact that the release curve slightly increases and decreases over time might be due to readsorption of the drug into the particles due to the static conditions used. The seemingly higher release rate at lower pH is due to the PDA coating. It has been observed that the PDA coating detaches and is partially peeled off from the surface of MSNs in acidic conditions. Thus, PDA works as "gate-keepers" and blocks the pores under neutral conditions, and owing to its pH-sensitivity the drug molecules are released under acidic conditions [2-3]. DOX also has a higher hydrophilicity and solubility at lower pH caused by increased protonation of NH_2 groups on DOX, which also most likely promotes the release at lower pH. The sustained release achieved with PDA coating is due to π-π stacking interactions with a high loading

strength [1]. This makes the PDA coated MSNs good candidates for sustained or even controlled drug delivery, depending on the design of the system. The difference in release behavior between PDA-coated and non-coated MSNs are more distinct for the rod-shaped MSNs than the spherical MSNs. This may be due to that for the S-MSN@PDA particles, the PDA-coating effect is more pronounced (as supported by a larger fraction of the pores being capped by PDA than for the R-MSN@PDA) while for R-MSN@PDA, the diffusion effect is more pronounced. From a structural point of view, Zhang, L. and his colleagues (2008) found that aspirin loaded into spherical MSNs had a faster release rate in PBS than aspirin loaded into rod-shaped MSNs, where the longer the rod-shaped MSNs were, the slower the release rate was due to longer and more twisted diffusion channels [5]. This indicates that structural differences of MSNs, tested media, and different hydrophobic/hydrophilic properties and functional groups of drug molecules affect the drug release rate from MSNs [4-5].

Calibration curve of Doxorubicin

The absorbance of supernatant of the particles before and after loading with DOX was measured at 480 nm using a UV-Vis spectrophotometer. A calibration curve (Figure S4) was done in order to facilitate the calculation of the amount of DOX loaded into the particles. A calibration with the R^2-value of 0.9992 was obtained, indicating that the data is linear. The equation obtained with the calibration curve was used for calculating the loading degree of DOX in DOX-loaded particles.

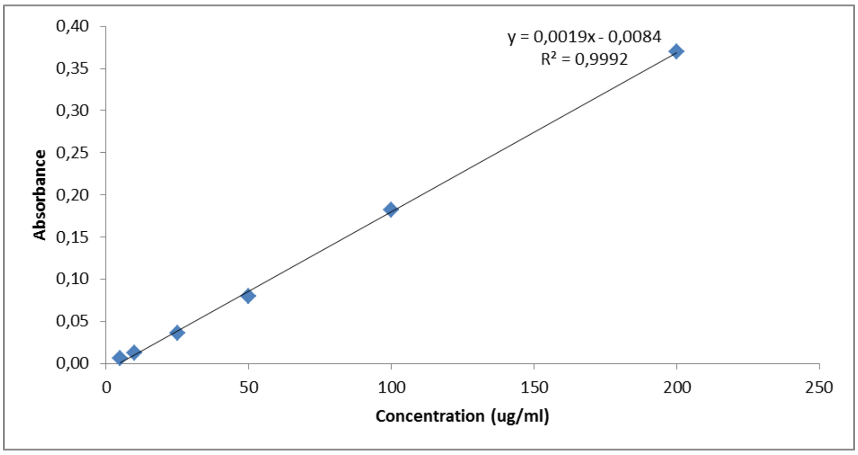

Figure S9. Calibration curve of doxorubicin in HEPES buffer.

References:

1. Zheng X, Zhang J, Wang J, Qi X, Rosenholm J and Cai K (2015). Polydopamine Coatings in Confined Nanopore Space: Toward Improved Retention and Release of Hydrophilic Cargo. *The Journal of Physical chemistry*, 119:24512-24521
2. Zheng Q, Lin T, Wu H, Guo L, Ye P, Hao Y, Guo Q, Jiang J, Fu F and Chen G (2014). Mussel-inspired polydopamine coated mesoporous silica nanoparticles as pH-sensitive nanocarriers for controlled release. *International Journal of Pharmaceutics,* 463:22-26
3. Chang D, Gao Y, Wang L, Liu G, Chen Y, Wang T, Tao W, Mei L, Huang L and Zeng X (2016). Polydopamine-based surface modification of mesoporous silica nanoparticles as pH-sensitive drug delivery vehicles for cancer therapy. *Journal of Colloid and Interface Science,* 463:279-287
4. Hao N, Li L and Tang F (2016). Shape matters when engineering mesoporous silica-based nanomedicines. *Biomaterial Science,* 4:575-591
5. Zhang L, Qiao S, Jin Y, Cheng L, Yan Z and Lu Q (2008). Hydrophobic Functional Group Initiated Helical Mesostructured Silica for Controlled Drug Release. *Advanced Functional Materials,* 18:3834-3842
